# Molecular characterization and differential expression of cytokinin-responsive type-A response regulators in rice (*Oryza sativa*)

**DOI:** 10.1186/1471-2229-6-1

**Published:** 2006-02-13

**Authors:** Mukesh Jain, Akhilesh K Tyagi, Jitendra P Khurana

**Affiliations:** 1 Interdisciplinary Centre for Plant Genomics and Department of Plant Molecular Biology, University of Delhi South Campus, Benito Juarez Road, New Delhi-110021, India

## Abstract

**Background:**

The response regulators represent the elements of bacterial two-component system and have been characterized from dicot plants like *Arabidopsis *but little information is available on the monocots, including the cereal crops. The aim of this study was to characterize type-A response regulator genes from rice, and to investigate their expression in various organs as well as in response to different hormones, including cytokinin, and environmental stimuli.

**Results:**

By analysis of the whole genome sequence of rice, we have identified ten genes encoding type-A response regulators based upon their high sequence identity within the receiver domain. The exon-intron organization, intron-phasing as well as chromosomal location of all the RT-PCR amplified rice (*Oryza sativa*) response regulator (*OsRR*) genes have been analyzed. The transcripts of *OsRR *genes could be detected by real-time PCR in all organs of the light- and dark-grown rice seedlings/plants, although there were quantitative differences. The steady-state transcript levels of most of the *OsRR *genes increased rapidly (within 15 min) on exogenous cytokinin application even in the presence of cycloheximide. Moreover, the expression of the *OsRR6 *gene was enhanced in rice seedlings exposed to salinity, dehydration and low temperature stress.

**Conclusion:**

Ten type-A response regulator genes identified in rice, the model monocot plant, show overlapping/differential expression patterns in various organs and in response to light. The induction of *OsRR *genes by cytokinin even in the absence of *de novo *protein synthesis qualifies them to be primary cytokinin response genes. The induction of *OsRR6 *in response to different environmental stimuli indicates its role in cross-talk between abiotic stress and cytokinin signaling. These results provide a foundation for further investigations on specific as well as overlapping cellular functions of type-A response regulators in rice.

## Background

Cytokinins regulate various plant growth and developmental processes, including cell division, apical dominance, chloroplast biogenesis, leaf senescence, vascular differentiation, photomorphogenic development, shoot differentiation in tissue cultures and anthocyanin production, primarily by altering the expression of diverse genes [1,2]. The recent genetic and molecular studies in plants have suggested the involvement of two-component sensor-regulator system in cytokinin signal perception and transduction, comprising sensor histidine kinase (HK) proteins, histidine phosphotransfer (HPt) proteins, and effector response regulator (RR) proteins [3-9]. Such signal transduction systems, once thought to be restricted to prokaryotes, have also been found in many eukaryotes, including yeast, fungi, slime molds and higher plants [10]. In *Arabidopsis*, proteins with homology to all the elements of two-component system have been identified [7].

The analysis of *Arabidopsis *genome revealed the existence of 32 putative response regulator genes [7]. Based on the predicted protein domain architecture and amino acid composition, the response regulators have been broadly categorized into three distinct families: type-A, type-B and pseudo-response regulators. The type-A response regulators are relatively small, containing a receiver domain along with small N- and C-terminal extensions [11]. The type-B response regulators comprise a receiver domain fused to the DNA-binding domain and are supposed to be transcriptional regulators [12-14]. The pseudo-response regulators share significant sequence similarity with the receiver domain of other response regulators but the invariant D-D-K motif is not present [7]. The pseudo-response regulators are also considered to be the elements of the circadian clock in *Arabidopsis *and rice [15-18].

The type-A response regulator genes in *Arabidopsis *(type-A *ARRs*) are rapidly and specifically induced by exogenous cytokinin, although with varying kinetics, and have been characterized as primary cytokinin response genes [11,19,20]. The transcription of type-A *ARR *genes is regulated in part by type-B ARRs [21,22]. Some of the type-A ARRs perform partially redundant functions, acting as negative regulators of cytokinin responses by a feedback mechanism [21,23,24]. In contrast, ARR4 was claimed to be a positive regulator of cytokinin signaling because its over-expression enhanced the cytokinin responsiveness of transgenic *Arabidopsis *plants [25]. However, the loss-of-function mutant did not reveal a positive role for ARR4 in cytokinin signaling [24] and this discrepancy remains to be resolved. The tissue distribution of ARR4 overlaps to a large extent with that of phytochrome B (phyB) and it has been found to interact with N-terminus of phyB to stabilize its active form [26]. The transgenic *Arabidopsis *plants overexpressing ARR4 are specifically hypersensitive to red light [26], indicating that ARR4 may be involved in integrating red light and cytokinin signaling.

The type-A response regulators have been isolated and characterized from maize [27,28]. However, there is no report on the characterization of any type-A response regulator from other monocot species, although several EST/cDNA sequences are available in the databases. Here, we report the identification and analysis of type-A response regulator gene family in rice (*Oryza sativa*), the model monocot plant. The exon-intron organization, chromosomal distribution and sequence homology have been analyzed for all ten members. The *OsRR *genes express differentially in various organs examined, and also in response to light. The application of exogenous cytokinin induced *OsRR *genes in the absence of *de novo *protein synthesis. Evidence has also been provided for a probable role of OsRR6 in abiotic stress signaling.

## Results and discussion

### Identification of type-A response regulators in rice

The information on rice genomic sequence [29,30] provides a powerful tool to identify putative homologous proteins by database searches with genes of known function from other organisms. In an attempt to identify type-A response regulators in rice, the whole rice genome, dynamically translated in all reading frames, was analyzed employing TBLASTN search, using *Arabidopsis *type-A response regulator proteins as query. This search identified a total of ten genes showing high sequence similarity with the *Arabidopsis *type-A response regulators. These predicted proteins contain a characteristic receiver domain (harboring conserved D-D-K residues) with short N- and C-terminal extensions and were designated as *Oryza sativa *response regulator (OsRR) proteins. We amplified (by RT-PCR) and sequenced nine *OsRR *cDNAs (*OsRR9 *and *10 *could not be amplified specifically since they possess >99% identity within the coding region and flanking sequences). Their sequences were confirmed by comparison with the corresponding genomic sequences. The cDNA sequences have been deposited in the GenBank, and their accession numbers, predicted protein length and genomic locus are given in Table 1.

### Exon-intron organization and chromosomal distribution

**Figure 1:**
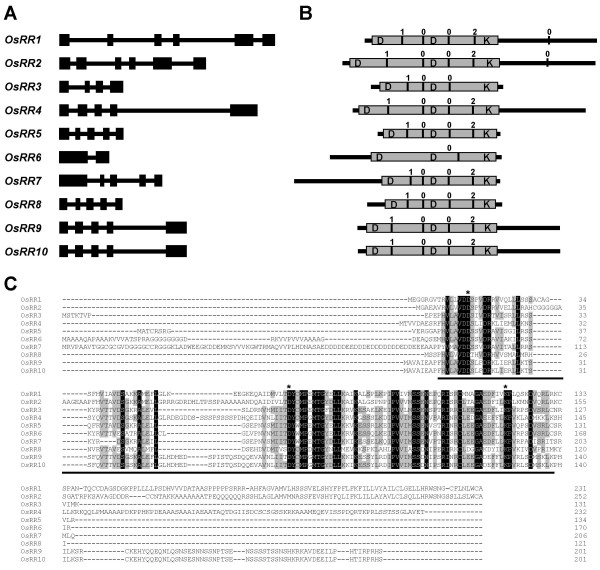
Type-A response regulators in rice. (A) Exon-intron organization of *OsRR *genes. Exons and introns are represented by black boxes and lines, respectively. (B) Schematic representation of OsRR proteins (aligned with respect to the second conserved Asp (D) residue). The conserved receiver domain is represented as gray box with D-D-K residues. The black vertical bars represent intron position. The numbers 0, 1 and 2 above the vertical bars represent the phase 0, 1 and 2 introns, respectively. (C) Multiple alignments of the OsRR proteins obtained with ClustalX. Fully and partially conserved (present in more than 50% of aligned sequences) residues are highlighted in black and gray boxes, respectively. Gaps (marked with dashes) have been introduced to maximize the alignments. The conserved receiver domain has been underlined. Conserved Asp and Lys residues are marked with asterisks.

A comparison of the full-length cDNA sequences (obtained in the present study) with the corresponding genomic DNA sequences (available in the database) showed that the coding sequence of majority of the *OsRR *genes (6 out of 10) are disrupted by 4 introns (Table 1, Fig. 1A), suggesting their origin from a common ancestral gene with a classical pattern of 5 exons and 4 introns. However, variations in their basic gene structure were observed for other members, implicating gain (*OsRR1 *and *OsRR2*) or loss (*OsRR3 *and *OsRR6*) of introns. The highly conserved intron phasing and the position of introns with respect to their amino acid sequences (Fig. 1B) also indicate their evolution from the same ancestral gene by exon shuffling [31].

The BAC (bacterial artificial chromosome) or PAC (phage artificial chromosome) clones carrying the genes for OsRR proteins were identified (Table 1). The chromosome map positions of BAC/PACs given in centiMorgans (cM) from top of the chromosome, and the nearest marker to each *OsRR *gene are indicated in Table 1. The ten *OsRR *genes were found to be distributed on 7 of the 12 rice chromosomes (Table 1). Three *OsRR *genes are present on chromosome 4, two on chromosome 2, and one each on chromosome 1, 7, 8, 11, and 12. The distribution of *OsRR *genes on rice chromosomes did not reveal evident clusters. However, *OsRR9 *and *OsRR10 *are present on the duplicated block between chromosome 11 and 12 [32,33].

### Sequence analysis

The type-A RRs are mainly composed of a receiver domain with short N- and C-terminal extensions [34], essentially similar to the *E. coli *response regulator (RR) CheY involved in chemotaxis, and lack a typical output domain. All the OsRR proteins also contain the highly conserved Lys and two Asp residues (D-D-K) in the receiver domain (Fig. 1B, C). Pairwise analysis of the full-length OsRR protein sequences indicates that the overall identities range from 28% to 71% (100% between OsRR9 and 10, Table 2). However, the amino acid identity within the receiver domain reaches up to 95%. The receiver domain of type-A RRs in *Arabidopsis *also showed 60% to 93% identity among them but less than 30% with that of type-B RRs. The predicted amino acid sequences of N- and C-terminal extensions of OsRR proteins are more variable (Fig. 1C), like *Arabidopsis *RR proteins [11]. The C-terminal extensions of ARR6 and ARR7 were shown to be responsible for their nuclear localization [35]. The rice type-A response regulators, OsRR1, OsRR2, OsRR4, OsRR9 and OsRR10, also possess C-terminal extensions rich in acidic and charged residues (Fig. 1B,C). However, OsRR6 and OsRR7 have N-terminal extensions rich in gly and asp residues (Fig. 1C). These N- and C-terminal variable regions may play a role in their localization to different cellular compartments.

**Figure 2:**
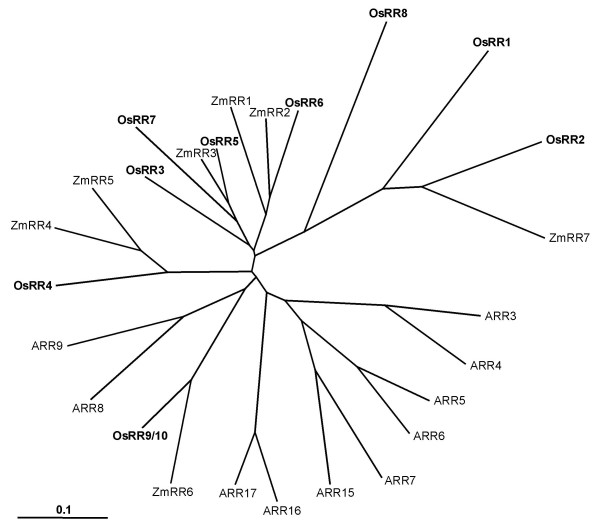
Phylogenetic relationship among type-A response regulator proteins from rice (OsRR), maize (ZmRR), and *Arabidopsis *(ARR). The unrooted tree was generated using ClustalX program by neighbor-joining method and visualized by Treeview. Scale bar represents 0.1 amino acid substitution per site.

To examine the phylogenetic relationships of rice, maize, and *Arabidopsis *type-A response regulators, an unrooted tree was constructed from alignments of their full-length protein sequences (Fig. 2). The OsRR proteins formed four sister pairs (OsRR1 and 2, OsRR3 and 6, OsRR5 and 7, OsRR9 and 10). This pairing of type-A RRs is consistent with recent evolutionary duplications postulated to have occurred in the rice genome [32,36]. The type-A response regulators in *Arabidopsis *also formed five sister pairs and all of them were found to be located on homologous duplicated chromosomal segments [24]. Thus, it is remarkable that the duplication of the sister pairs of type-A response regulator genes is associated with chromosomal block duplications in both rice and *Arabidopsis*. Recently, the extensive duplication and preferential retention of early auxin-responsive *Aux/IAA *genes have been reported in both *Arabidopsis *and rice [37,38]. The retention of the duplicated type-A response regulator genes in rice (present study) also supports the idea that the genes involved in transcription and signal transduction have been preferentially retained in *Arabidopsis *[39]. Also, most of the OsRRs clustered together alongwith maize RRs (ZmRRs), in clades distinct from *Arabidopsis *RRs (ARRs) (Fig. 2). In an earlier study with *Arabidopsis *RRs, a phylogenetic analysis, including only a few of the monocot RRs, revealed that the type-A ARRs fall into clades distinct from rice and maize [24]. The present study corroborates this observation and strengthens the view that the progenitor of monocots and dicots probably had only a small family of RRs and the expansion occurred subsequently, in both monocots and dicots, by gene duplication.

### Organ-specific expression of *OsRR *genes

**Figure 3:**
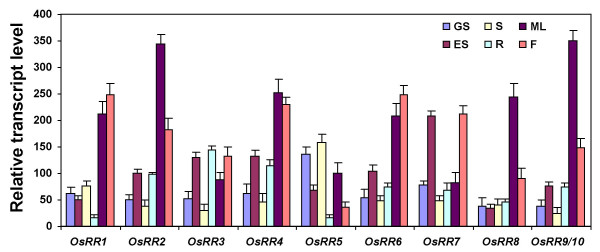
Real-time PCR analysis showing the organ-specific expression profiles of individual *OsRR *genes. The relative mRNA levels of individual *OsRR *genes normalized with respect to housekeeping gene, *UBQ5*, in different tissues (GS, green seedlings; ES, etiolated seedlings; S, green shoots; R, roots; ML, mature leaves; F, flowers) are presented.

To examine the expression pattern of each *OsRR *gene in different plant organs, and to assess the effect of light, quantitative real-time PCR analysis was performed with total RNA isolated from etiolated seedlings, green seedlings, green shoots, roots, mature leaves, and flowers. The transcripts of all the *OsRR *genes were detected in various organs examined but they displayed a complex expression pattern (Fig. 3). Most of the *OsRR *genes were expressed at relatively higher level in mature tissues (leaves and flowers). The expression of *OsRR *genes was also higher in roots (present study), which is essentially similar to most of the ARRs [11]. The transcript levels of *OsRR2*, *3*, *4*, *6*, *7*, and *9 *were significantly higher in etiolated seedlings as compared to green seedlings (Fig. 3). However, *OsRR5 *exhibited higher expression in green seedlings. Interestingly, in a recent study, the *arr *mutants in *Arabidopsis *exhibited altered red light sensitivity [24]. Also, ARR4 has been found to interact with phytochrome B and stabilize its active form [26]. These results suggest that *OsRR *genes exhibit overlapping and differential expression patterns, playing predominant roles in specific organs and may also be involved in light signal transduction.

### *OsRRs *represent primary cytokinin response genes

**Figure 4:**
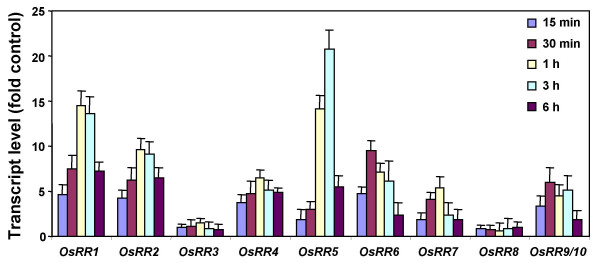
Kinetics of induction of *OsRR *genes in response to cytokinin. The transcript levels of each *OsRR *gene in 6-day-old light-grown seedlings treated with BAP for 15 min, 30 min, 1 h, 3 h and 6 h, were plotted as the relative expression (fold) of the seedlings mock-treated for the same duration.

Cytokinin regulates the expression of a large number of genes including type-A response regulators in *Arabidopsis *and maize [11,27,40]. To study the effect of cytokinin on the transcript levels of individual *OsRR *genes, the light-grown rice seedlings were treated with exogenous cytokinin for various durations over a 6 h period, and real-time PCR analysis was performed. Consistent with the results reported earlier with *Arabidopsis *[11], the steady-state transcript levels of *OsRR *genes were elevated within 15 min of exogenous cytokinin application (Fig. 4). The kinetics of induction with cytokinin was similar for most *OsRR *genes, with the transcript levels reaching its maximum in 1 h, and declining thereafter. There was, however, no significant change in the transcript abundance of *OsRR3 *and *8 *following cytokinin treatment (Fig. 4). The rapid induction of type-A ARRs by exogenous cytokinin has been shown to mediate a feedback mechanism, which decreases the sensitivity of the plant to the hormone, indicating that type-A RRs act as negative regulators of cytokinin-induced responses [21,23,24]. The type-B RRs are the transcription factors that are involved in the transcriptional activation of the type-A response regulators in response to cytokinins [13,21,22].

**Figure 5:**
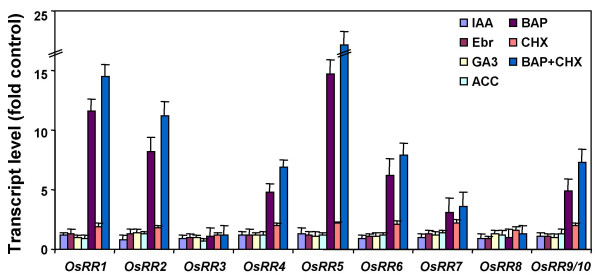
Expression profiles of the *OsRR *genes in response to different hormones and cycloheximide. The transcript levels of each *OsRR *gene in 6-day-old light-grown seedlings treated with IAA, Ebr, GA3, ACC, BAP, CHX and CHX+BAP for 3 h, were plotted as the relative expression (fold) of the seedlings mock-treated for the same duration.

Many primary response genes, such as auxin-induced *Aux*/*IAA *genes, are induced in the absence of *de novo *protein synthesis [41]. The genes encoding type-A RRs in *Arabidopsis *have also been described as the primary cytokinin response genes [11,42]. To determine whether the *OsRR *genes also belong to the same category, their induction by cytokinin was examined in the presence of a protein synthesis inhibitor, cycloheximide (Fig. 5). The steady-state transcript levels of most of the *OsRR *genes increased (up to 2- to 2.5-fold) on treatment with cycloheximide, suggesting that the transcription of these genes is regulated by a short-lived repressor protein. The increase in transcript abundance (up to 4- to 6-fold) of rice *Aux/IAA *genes examined (*OsIAA9*, *OsIAA13 *and *OsIAA20*) [38] in the same RNA samples (data not shown), confirmed that cycloheximide treatment was biologically effective in our experiments. Further, cycloheximide failed to block the induction of these genes by cytokinin; the transcript levels of *OsRR *genes were higher in the samples treated with BAP in the presence of cycloheximide as compared to BAP alone (Fig. 5). These results suggest that *OsRR *genes constitute a class of primary cytokinin response genes.

The expression of *OsRR *genes in response to other plant hormones, including auxin, brassinosteroid, gibberellin and ethylene, was also examined. However, no significant effect of these hormones could be detected on the steady-state transcript levels of *OsRR *genes (Fig. 5), indicating their role primarily in cytokinin signaling.

### Expression of *OsRR *genes under stress conditions

**Figure 6:**
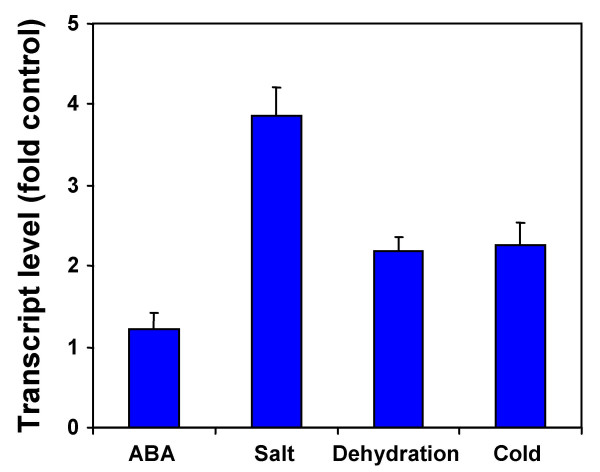
Changes in transcript levels of the *OsRR6 *gene in response to different stress treatments. The transcript levels of *OsRR6 *gene in 6-day-old light-grown seedlings treated with ABA, NaCl, mannitol and cold for 6 h, were plotted as the relative expression (fold) of the seedlings mock-treated for the same duration.

It has been claimed earlier that the expression of some of the type-A response regulators in *Arabidopsis *is induced by different environmental stresses such as drought, salinity and low temperature [43]. To investigate, whether some of the *OsRR *genes are also involved in stress responses, their expression levels were examined by real-time PCR analysis of the RNA isolated from young rice seedlings subjected to various abiotic stress treatments. The expression of a majority of *OsRR *genes was not significantly altered under stress (data not shown), with the notable exception of *OsRR6*. The expression of *OsRR6 *gene was induced to significant levels by salt, dehydration and low temperature treatments (Fig. 6), and results were reproducible. This indicates that OsRR6 may play an important role in abiotic stress signaling in rice, besides acting as a component in cytokinin signaling. It is noteworthy here that a transmembrane hybrid-type histidine kinase, AHK1, closely related to cytokinin receptors, is a putative osmosensor in *Arabidopsis *[44].

### What are the probable functions of OsRR proteins?

The type-A RRs in *Arabidopsis *have been shown to act as the negative regulators of cytokinin signaling with partially redundant functions [11,24]. In addition, some of these ARRs and other elements of the two-component sensor-regulator phosphorelay participate in cross-talk between cytokinin and light signaling (mediated by phytochromes) and also with the gaseous hormone ethylene [4,45]. The role of one of the type-A RRs, i.e. ARR4, has also been ascribed in red light mediated photomorphogenesis [24,26]. In fact, ARR4 physically associates with phytochrome B to prolong the stability of its active conformation, Pfr, and accentuates red light signaling. The role of type-B ARRs (and possibly of type-A ARRs) has also been envisaged in a cross-talk between ethylene and cytokinin signaling, although they may regulate these components differentially to control diverse plant processes [4]. To have an inkling about the functions of *OsRR *genes, the rice *Tos17 *insertion mutant database [46] providing the phenotype of rice *Tos17 *retrotransposon insertion mutants [47] was accessed using the BLAST program. We could identify insertion mutants corresponding only to *OsRR9/10 *gene (NE6006_0_401_1A and ND8005_0_402_1A); it was difficult to demarcate whether the sequence flanking the *Tos17 *insertion represents *OsRR9 *or *OsRR10 *because of more than 99% similarity between them. Despite the fact that *OsRR9 *and *OsRR10 *genes are so similar and may function redundantly, the phenotype of the insertion mutants (representing only one of these two loci) showed dwarfism, sterility, lesion mimic and vivipary. It can thus be speculated that *OsRR9/10 *genes may quantitatively affect different cellular processes influenced by both light and cytokinin. A detailed analysis of the insertional mutants already available and RNAi strategy for the remaining *OsRR *genes will greatly help in elucidation of the precise role of these genes.

## Conclusion

The results of structural analyses of rice type-A RR proteins and their phylogenetic relationship with ARRs will be helpful for their functional validation in rice. The organ-specific differential expression profile of *OsRR *genes suggests that their products most likely perform diverse and overlapping functions in different cell types of rice. This study also reflects the role of *OsRR *genes in both light and cytokinin signaling. The induction of *OsRR6 *by different abiotic stress stimuli provides a molecular link between stress and cytokinin signaling as well. These results provide a foundation for future work on the elucidation of cellular functions of type-A response regulators in rice.

## Methods

### Sequence analysis

The type-A response regulator genes in rice were identified by performing BLAST searches at the National Centre for Biotechnology Information [48] and TIGR genomic and annotated database [49] resources of rice using the response regulator protein sequences of *Arabidopsis thaliana *as query. The number and positions of exons and introns for individual *OsRR *genes were determined by comparison of the cDNAs with their corresponding genomic DNA sequences. The position of each gene on rice chromosomes was found by BLASTN search in genomic sequences of rice chromosome pseudomolecules available at TIGR (Release 3). Multiple sequence alignments were done using the ClustalX (version 1.83) program [50] and the phylogenetic analysis carried out by neighbor-joining method [51]. The unrooted phylogenetic tree was displayed using the Treeview program. The DNA and protein sequence analyses were performed using Gene Runner program version 3.04. Pairwise comparisons were done with the DNASTAR MegAlign 4.03 package to determine the sequence identities.

### Plant material and growth conditions

Rice (*Oryza sativa *L. ssp. *indica *var. Pusa Basmati 1) seeds were disinfected with 0.1% HgCl_2 _solution for 1 h and thoroughly washed with RO (reverse-osmosis) water before soaking overnight in RO water. Seedlings were grown on cotton saturated with RO water, at 28 ± 1°C, either in complete darkness or in a culture room with a daily photoperiodic cycle of 14 h light and 10 h dark. Flowers and mature leaves were collected from rice plants grown in the greenhouse.

### Hormone and stress treatments

For cytokinin treatment, 6-day-old light-grown rice seedlings were transferred to a solution of 50 μM benzyl aminopurine (BAP) and harvested at the indicated times. For treatment with other hormones/compounds, 6-day-old light-grown rice seedlings were transferred to beakers containing solutions of indole-3-acetic acid (IAA, 50 μM), epibrassinolide (Ebr, 10 μM), gibberellic acid (GA3, 50 μM), 1-aminocyclopropane-1-carboxylic acid (ACC, 50 μM), abscisic acid (ABA, 50 μM) and cycloheximide (CHX, 50 μM) and incubated for for 3 h. The mock-treated seedlings for the respective time intervals served as the control.

For salt and drought stress treatments, 6-day-old light-grown rice seedlings were transferred to 250 mM NaCl or 300 mM mannitol for 6 h. For low temperature treatment, the seedlings were kept at 8 ± 1°C for 6 h. The seedlings kept in water for the same duration, and at 28 ± 1°C, served as control.

### RNA isolation

Total RNA was extracted using the RNeasy Plant mini kit (Qiagen, Germany). To remove any genomic DNA contamination, the RNA samples were treated with RNase-free DNase I (Qiagen) according to the manufacturer's instructions. For each RNA sample, absorption at 260 nm was measured and RNA concentration calculated as A_260 _× 40 (μg/mL) × dilution factor. The integrity of RNA samples was monitored by agarose gel elecrophoresis.

### cDNA isolation, cloning and sequencing

The coding region of *OsRR genes *were amplified by RT-PCR, using gene-specific primers, from total RNA isolated from light-grown seedlings using Titan One Tube RT-PCR kit (Roche, USA) according to the manufacturer's instructions. After 30 or 35 cycles, the PCR products were examined by gel electrophoresis and EtBr staining. RT-PCR products were cloned into pGEM-T Easy vector (Promega, Madison, WI) as per manufacturer's instructions. Sequencing was carried out using ABI Prism 377 Sequencer (PE Applied Biosystems, USA), with the Thermosequenase Dye Terminator Cycle Sequencing Kit (Amersham, UK).

### Quantitative real-time PCR expression analysis

The transcript levels of type-A *OsRR genes *in various RNA samples were quantified by real-time PCR analysis employing ABI Prism 7000 Sequence Detection System and software (PE Applied Biosystems, USA) as described previously [52]. Primers were designed by using the primer design software Primer Express 2.0 (PE Applied Biosystems, USA). To ensure that the primers amplify a unique and desired cDNA segment, each pair of primers was checked in the BLAST program in rice genomic sequence available in TIGR database. The primer sequences are listed in Table 3. Primers specific to *OsRR9 *and *10 *could not be designed as more than 99% identity was found in the coding region and flanking sequences. First strand cDNA was synthesized by reverse transcribing 3 μg of total RNA using High Capacity cDNA Archive kit (Applied Biosystems, USA) according to the manufacturer's instructions. Diluted cDNA samples were used as template and mixed with 200 nM of each primer and SYBR Green PCR Master Mix (Applied Biosystems, USA) for real-time PCR analysis. PCR reactions were performed using the following parameters: 2 min at 50°C, 10 min at 95°C, and 40 cycles of 15 s at 95°C and 1 min at 60°C in 96-well optical reaction plates (Applied Biosystems, USA). The identities of the amplicons and the specificity of the reaction were verified by agarose gel electrophoresis and melting curve analysis, respectively. The relative mRNA levels for each of the *OsRR *genes in different RNA samples were computed with respect to the internal standard, *UBQ5*, to normalize for variance in the quality of RNA and the amount of input cDNA. At least two different RNA isolations and cDNA syntheses were used for quantification and each cDNA sample subjected to real-time PCR in triplicate. The values presented are the mean of the two biological replicates, each with three technical replicates. The error bars indicate the standard deviation from the mean.

## Authors' contributions

MJ designed and performed all the *in silico *analysis and wet-lab experiments, and drafted the manuscript. AKT and JPK participated in design of experiments, coordinated the study, and helped to draft the manuscript. All authors read and approved the final manuscript.

## Figures and Tables

**Table 1:** Type-A response regulators in rice.

**Gene name^a^**	**Accession no.^b^**	**ORF Length (bp)^c^**	**Polypeptide length (aa)^d^**	**No. of introns^e^**	**Chromosome no.^f^**	**Genomic locus^g^**			**Nearest marker^h^**
							
						**BAC/PAC name**	**Accession no.**	**cM position**	
*OsRR1*	AJ938070	696	231	5	4	P0076O17	BX548156	65.3	E60696S
*OsRR2*	AJ938071	759	252	5	2	OSJNBb0038F20	AP005808	81.7–83.6	C499
*OsRR3*	AJ938072	396	131	3	2	OJ1124_D06	AP004043	157.9	C1470
*OsRR4*	AJ938073	699	232	4	1	P0431G06	AP003683	169.5	S10581
*OsRR5*	AJ938074	405	134	4	4	OSJNBb0065J09	AL663010	78.2–81.7	C1558S
*OsRR6*	AJ938075	513	170	1	4	OSJNBb0004A17	AL606652	123.8	R2231
*OsRR7*	AJ938076	621	206	4	7	OJ1047_A06	AP003802	53.4	C735
*OsRR8*	AJ938077	366	121	4	8	OJ1705_C03	AP003962	60.1–60.4	E61839S
*OsRR9*	AJ938078	606	201	4	11	OSJNBa0059J06	AC123526	8.1–8.6	R642A
*OsRR10*	AJ938078	606	201	4	12	OSJNBb0034E23	AL928752	12.2	R642B

^a ^Systematic designation given to rice type-A response regulator genes. ^b ^cDNA Genbank accession number (*OsRR9 *and *10 *has been assigned the same accession number as they could not be amplified specifically). ^c ^Length of open reading frame in base pairs. ^d ^Length (number of amino acids) of the deduced polypeptide. ^e ^Number of introns present within ORF. ^f ^Chromosomal localization of *OsRR *gene. ^g ^Name, accession number and approximate cM position of the BAC/PAC clone in which the corresponding *OsRR *gene is present. ^h ^Nearest marker to the *OsRR *gene.

**Table 2:** Percentage identities among the rice type-A response regulators.

	OsRR1	OsRR2	OsRR3	OsRR4	OsRR5	OsRR6	OsRR7	OsRR8	OsRR9	OsRR10
OsRR1	-	63.2/53.7	44.4/44.3	33.6/28.6	47.7/47.0	36.1/35.3	29.1/27.7	34.4/38.8	34.8/29.9	34.8/29.9
OsRR2		-	43.9/45.0	35.9/32.3	47.7/45.5	40.2/30.6	34.8/26.7	36.6/38.8	38.8/29.4	38.8/29.4
OsRR3			-	53.5/55.7	66.7/71.0	64.6/67.9	60.6/62.6	44.8/48.8	51.3/55.0	51.3/55.0
OsRR4				-	57.0/56.0	41.5/45.3	32.2/29.1	46.7/44.6	58.1/55.7	58.1/55.7
OsRR5					-	67.4/72.4	67.4/68.7	50.0/51.2	54.3/55.2	54.3/55.2
OsRR6						-	55.8/53.5	38.5/47.9	42.5/43.5	42.5/43.5
OsRR7							-	44.3/43.8	31.8/29.4	31.8/29.4
OsRR8								-	43.7/43.8	43.7/43.8
OsRR9									-	99.4/100.0
OsRR10										-

Values indicate the percentage identity obtained by pairwise comparisons between ORF (left) and protein sequences (right).

**Table 3:** Primer sequences used for real time PCR expression analysis.

**Gene name**	**Primer sequence**
*OsRR1*	5'-AGGATCAGCAGATGCATGAATG-3'
	5'-GAGACGCTGTACGTCCTTGCTT-3'
*OsRR2*	5'-ACGATCTTCTCAAAGCCATCAAG-3'
	5'-TGAGAGGCTTAAGGATGAAATCCT-3'
*OsRR3*	5'-CAGGGTTCGATCTCCTCAAGAG-3'
	5'-CGAATTCTCCGACGACATTAGC-3'
*OsRR4*	5'-TCTTCTGAGAATGTGCCTGCAA-3'
	5'-GCTTGACAGGTTTCAGGAAGAACT-3'
*OsRR5*	5'-ACCGAATGTGAGCATGATTATCA-3'
	5'-CCTTGACCTTCTTCAGGAGTTCATA-3'
*OsRR6*	5'-GTCCCCAACGTCAACATGATC-3'
	5'-CACGTTCTCCGACGACATGAT-3'
*OsRR7*	5'-TGCTCAAGAAGATCAAGGAATCG-3'
	5'-GGCACGTTCTCTGACGACATTAT-3'
*OsRR8*	5'-CCAGACATGACCGGCTATAACC-3'
	5'-AAGCAATTACAACCGGGAGATG-3'
*OsRR9/10*	5'-TCATGAGGACAGCCCAATTTCTA-3'
	5'-TGCAGTAGTCTGTGATGATCAGGTT-3'
*UBQ5*	5'-ACCACTTCGACCGCCACTACT-3'
	5'-ACGCCTAAGCCTGCTGGTT-3'
